# Proteomic profile data of rabbit stem cell secretome and stem cell/tenocyte secretome and gene expression data, scratch assay data and CAM assay data of tenocyte in vitro cultures treated with these two different secretomes

**DOI:** 10.1016/j.dib.2025.111789

**Published:** 2025-06-17

**Authors:** Petra Wolint, Iris Miescher, Asma Mechakra, Patrick Jäger, Julia Rieber, Maurizio Calcagni, Pietro Giovanoli, Viola Vogel, Jess G. Snedeker, Johanna Buschmann

**Affiliations:** a Division of Surgical Research, University Hospital of Zurich, 8091 Zurich, Switzerland; b Department of Plastic Surgery and Hand Surgery, University Hospital Zurich, 8091 Zurich, Switzerland; c Institute for Biomechanics, ETH Zurich, 8092 Zurich, Switzerland; d Balgrist University Hospital, University of Zurich, 8008 Zurich, Switzerland; e Laboratory of Applied Mechanobiology, Department of Health Sciences and Technology, ETH Zurich, 8092 Zurich, Switzerland

**Keywords:** Angiogenesis, Proteomics, Secretome, Mesenchymal stem cells, Tenocytes, Tendon healing

## Abstract

Tendon rupture repairs exhibit two main problems. On the one hand side, they often lead to scar formation during healing, which has inferior mechanical properties and may lead to re-ruptures. On the other hand side, fibrotic adhesion formation to the surrounding tissue may occur, which hampers proper range of motion.

Cell-free approaches to support tendon healing are getting increasing attention worldwide. We have therefore obtained two different cell secretomes from New Zealand White rabbit adipose tissue-derived mesenchymal stem cells (ADSCs) or from a 3:1 mixed culture of ADSCs and rabbit Achilles tenocytes. These cell secretomes were examined and compared in terms of their proteomic profiles and biological activities. Applied to a tenocyte *in vitro* culture, gene expression analysis data and scratch assay data are provided. To determine the angiogenic potential, the chorioallantoic membrane of the chicken embryo (CAM) was treated with the secretomes.

## Graphical abstract

**Figure:**
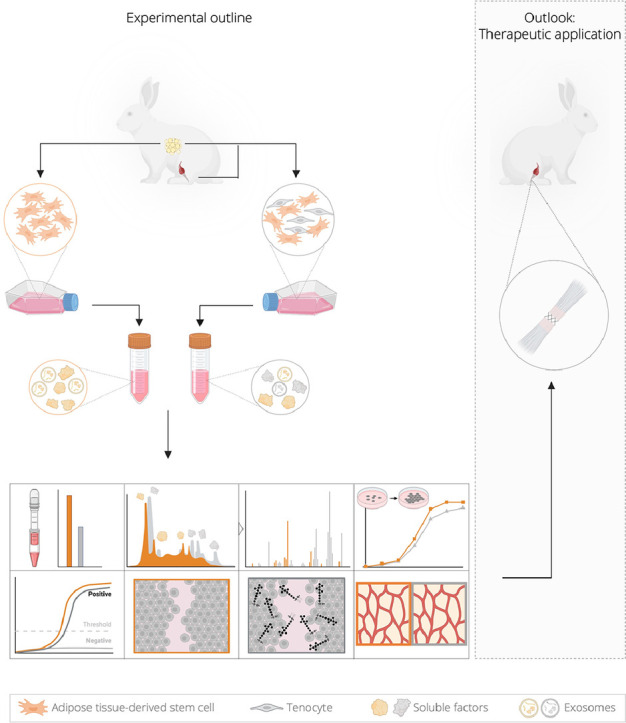

Specifications Table

**Table:** 

Subject	Angiogenesis, cell biology, proteomics, gene expression
Specific subject area	Two secretomes (either harvested from rbADSCs or from rbADSC:rbTenocytes co-culture (3:1) were analysed for their protein content and protein profile. They were applied to an rbTenocyte in vitro cell culture and gene expression data, scratch assay data as well as proliferation data were determined. Furthermore, the two secretomes were applied in the CAM assay in order to assess the angiogenic response.
Data format	The data consist of raw and analysed data.
Type of data	Excel files of the type *.xlsx* files.
Data collection	Data collection was performed with different kinds of experiments, including cell cultures of either rbADSCs or rbADSC:rbTenocytes in a 3:1 ratio; harvesting their secretomes – and application in rbTenocyte cell culture to assess impact on gene expression. Moreover, rbTenocytes were used for the scratch assay. Finally, we used the CAM assay to collect data on angiogenic readouts and to calculate the angiogenic activity index (AAI). As for the proteomics, we used a service facility (the Functional Genomics Center Zurich), where they measured protein composition of each secretome via LC–MS/MS. We have then analysed the proteomic profile for functional enrichment and for receptor-ligand interactions.
Data source location	Except for the proteomics, all data were collected in Zurich, Switzerland, at the University Hospital Zurich in the laboratories of the Department for Plastic Surgery and Hand Surgery. The proteomic profile was assessed in Zurich, Switzerland, at the Functional Genomics Center Zurich (FGCZ) of University of Zurich and ETH Zurich.
Data accessibility	**Repository name**: Mendeley Data **Data identification number**: 10.17632/gpzhzcpykp.1 **Direct URL** to data: Proteomic profile data of rabbit stem cell secretome and stem cell/tenocyte secretome and gene expression data, scratch assay and CAM assay data of tenocyte in vitro cultures supplemented with these two different secretomes - Mendeley Data
Related research article	Therapeutic Potential of Mesenchymal Stem Cell and Tenocyte Secretomes for Tendon Repair: Proteomic Profiling and Functional Characterization In Vitro and In Ovo

## 1 Value of the Data

• Our data can be reused, if other rabbit cell types than rabbit adipose-derived stem cells or rabbit tenocytes are used to harvest secretome.
• These data can be reused, if other species than rabbits are used for the extraction of ADSCs or tenocytes and to produce secretomes from them.
• These data can be compared with drugs that are supplemented to the scratch assay or to the CAM assay.
• Our proteomic data set can be re-analysed and re-used to elucidate signaling pathways that may appear interesting after the application of such secretomes to ruptured tendons.
• As these data were produced from single cell monolayer cultures, they can be reused for comparison with secretomes harvested from spheroids of any size.

## 2 Background

As tendons lack a proper vascularization and because tenocytes have a low metabolic activity, tendon ruptures heal only slowly, often with a suboptimal outcome: lower strength and reduced range of motion. Therefore, novel therapeutic approaches are welcome. Among them, particularly cell-free strategies are being investigated, where different growth factors, cytokines and other factors are intended to be applied that may support tendon healing [1].

Platelet rich plasma (PRP) has been used in a diverse set of conditions, however, the protocols to obtain PRP vary a lot among the studies [[2], [3], [4]]. Cell-free approaches include tendon stem/progenitor derived exosomes [5] or conditioned medium from ADSCs [6]. A comprehensive review discusses diverse cell-free strategies, with an emphasis on infrapatellar fat pad-derived non-cellular products [7].

Based on this background, we provide a comprehensive characterization data set for two differently produced rabbit cell secretomes and data on their biological impact, with respect to proliferation, gene expression, migration and angiogenesis.

## 3 Data Description

**Fig. 1:**
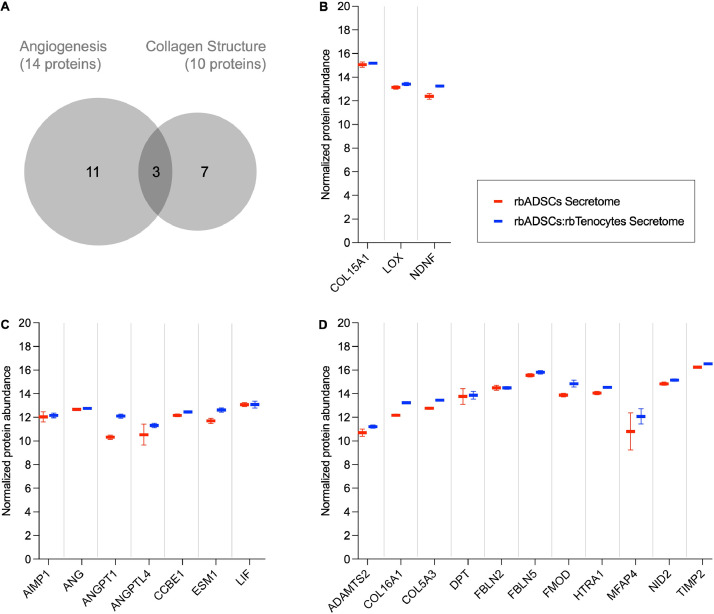
Protein expression detected in secretomes. The Venn diagram displays the overlap of certain selected expressed proteins in processes related to angiogenesis and collagen structure (A). The selection of proteins relevant to tendon development and healing is based on normalised protein abundance, showing proteins in the secretome obtained from rabbit adipose tissue derived mesenchymal stem cells (rbADSCs) and rbADSCs-rbTenocytes culture (ratio 3:1) that are relevant to both angiogenesis and collagen structure (B), angiogenesis only (C), and collagen structure only (D). Abbreviations: ADAMTS2 = ADAM metallopeptidase with thrombospondin type 1 motif 2, AIMP1 = aminoacyl TRNA synthetase complex interacting multifunctional protein 1, ANG = angiogenin, ANGPT1 = angiopoietin 1, ANGPTL4 = angiopoietin like 4, CCBE1 = collagen and calcium binding EGF domains 1, COL15A1 = collagen type XV alpha 1 chain, COL16A1 = collagen type XVI alpha 1 chain, COL5A3 = collagen type V alpha 3 chain, DPT = dermatopontin, ESM1 = endothelial cell specific molecule 1, FBLN2 = fibulin 2, FBLN5 = fibulin 5, FMOD = fibromodulin, HTRA1 = HtrA serine peptidase 1, LIF = LIF interleukin 6 family cytokine, LOX = lysyl oxidase, MFAP4 = microfibril associated protein 4, NDNF = neuron derived neurotrophic factor, NID2 = nidogen 2, TIMP2 = TIMP metallopeptidase inhibitor 2.

The data are stored as a set of Microsoft Excel files (Microsoft Corporation, Redmond, WA, USA) (.xlsx files) in a Mendeley Data repository service entitled Proteomic profile data of rabbit stem cell secretome and stem cell/tenocyte secretome and gene expression data, scratch assay and CAM assay data of tenocyte in vitro cultures supplemented with these two different secretomes - Mendeley Data. This repository contains five excel files, named DiB_Rabbit Secretomes_Figures2I_S1.xlsx; DiB_Rabbit Secretomes_Figures3_4_S2-4.xlsx; DiB_Rabbit Secretomes_Figure5.xlsx; DiB_Rabbit Secretomes_Figure6.xlsx; and DiB_Rabbit Secretomes_Figures7_S5.xlsx, respectively. The titles of these excel files contain the *Figure* number of the related research article, to assure where which of the raw data have been analysed to generate the corresponding figures [1].

### 3.1 Gene expression data of Figure 2I of the related research article

**File** DiB_Rabbit Secretomes_Figures2I_S1.xlsx

This excel file is open access published in Mendeley Data Proteomic profile data of rabbit stem cell secretome and stem cell/tenocyte secretome and gene expression data, scratch assay and CAM assay data of tenocyte in vitro cultures supplemented with these two different secretomes - Mendeley Data and contains 1 sheet, called *Fig2I S1*, according to the panel I in the related research article Fig. 2 (quantitative real time polymerase chain reaction with the comparative deltaCT method). The sheet *Fig2I S1* contains in column A the sample description, with cell type, donor and passage number (abbreviated with P), then in columns B-D, the name, the CT and the mean CT of the housekeeping gene, afterwards in column E and F, the name and the CT of the target genes, followed by column G with dCT and H with –dCT, and column I with mean –dCT and column J denoting the standard deviation (SD) of –dCT. Finally, in columns L and M, we provide all abbreviations that were used in this sheet *Fig2I S1.*

### 3.2 Protein concentration; normal abundance matrix, differential expression and enrichment analysis

**File** DiB_Rabbit Secretomes_Figures3_4_S2-4.xlsx

This excel file is open access published in Mendeley Data Proteomic profile data of rabbit stem cell secretome and stem cell/tenocyte secretome and gene expression data, scratch assay and CAM assay data of tenocyte in vitro cultures supplemented with these two different secretomes - Mendeley Data and contains 3 sheets, called *Fig3A, Fig 3B-D_4_S2_S4*, and *Fig3E-G_S3*, respectively, according to the panels in the related research article Fig. 3 [1].

The first sheet *Fig3A* contains the sample description in column A, the protein concentrations which were measured three times with each repetition in column B, C and D, respectively; the average protein concentration (mean) in column E, the standard deviation (SD) in column F, the unit in column G and the key for the abbreviations used in this sheet in column I and J.

The second sheet *Fig 3B-D_4_S2_S4* contains data on the normal abundances matrix (columns A-O); with protein ID (identification) in column A, with the description of the corresponding protein in column B, with the number of peptides in column C, with fasta ID in column D, the protein group 2 in column E, the ID column in column F, the CON and REV in columns G and H; the isotope label in I, the values for rbADSC-derived secretomes from donors 1-3 in columns J-L and the values for the rbADSC/Tenocyte-co-culture-derived secretomes 1-3 in columns M-O. This second sheet also contains the differential expression analysis in columns R-AK; including protein ID (identification) in column R, with the description of the corresponding protein in column S, with the number of peptides in column T, with *fasta ID* in column U, the protein group 2 in column V, the ID column in column W, the CON and REV in columns X and Y; furthermore, in column Z there is the model name, in column AA the contrast, in column AB the difference, in column AC the standard error, in column AD the average abundance, in column AE the statistics, in column AF the degree of freedom, in column AG the associated p-value, in column AH the lower confidence interval, in column AI the higher confidence interval, in column AJ the corresponding sigma value and in column AK the false discovery rate FDR. Finally, columns AN and AO define the abbreviations used before.

The third sheet *Fig3E-G_S3* contains data on differential enrichment analysis; with protein ID (identification) in column A, with the description of the corresponding protein in column B, with the number of peptides in column C, with *fasta ID* in column D, the protein group 2 in column E, the ID column in column F, the CON and REV in columns G and H; furthermore, in column I there is the model name, in column J the contrast, in column K the difference, in column L the standard error, in column M the average abundance, in column N the statistics, in column O the degree of freedom, in column P the associated p-value, in column Q the lower confidence interval, in column R the higher confidence interval, in column S the corresponding sigma value and in column T the false discovery rate FDR, in column U the symbol of the corresponding gene, in column V the logarithm on basis 10 of the FDR value, in column X the list of *upregulated* proteins, in column Y the symbol of the ligand gene and in column Z the symbol of the receptor gene. Finally, columns AC-AE reflect the same content as columns X-Z do, however, for *downregulated* proteins.

As for proteins related to angiogenesis and collagen structure, we have selected some data that are shown in Fig. 1.

### 3.3 Gene expression data of Figure 5 of the related research article

**File** DiB_Rabbit Secretomes_Figure5.xlsx

This excel file is open access published in Mendeley Data Proteomic profile data of rabbit stem cell secretome and stem cell/tenocyte secretome and gene expression data, scratch assay and CAM assay data of tenocyte in vitro cultures supplemented with these two different secretomes - Mendeley Data and contains 13 sheets, called *IL6, MMP9, Timp1, Ki67, ALOX15, aSMA, BGN, TNC, MKX, COL3A1, COL1A1, MMP2* and *TNMD*, respectively. As all sheets are structured the same, the description will be done only for the first sheet *IL6*. Column A describes the cell type, rabbit donor, the passage (for example P3 is passage 3), and the treatment – with medium as the control, and the two secretome treatments; either rbADSC secretome or rbADSC:rbTenocytes secretome. Column B then indicates the time point in days. Furthermore, columns C-E refer to the reference gene, the so-called housekeeping gene, with its name (*18S*), the CT and the CT mean values. Columns F and G represent the name and CT values of the target genes, so for this first sheet *IL6*. Column H calculates the deltaCT value (*dCT*), while columns I, J and K show the mean of dCTs, the deltadeltaCTs (*ddCT*) and the 2^−ddCT^ values. Columns L and M give means and standard deviations of 2^−ddCT^ values. Abbreviations and colors used in this sheet are explained in the key in columns O and P, respectively.

### 3.4 Gene expression data of Figure 6 of the related research article

**File** DiB_Rabbit Secretomes_Figure6.xlsx

This excel file is open access published in Mendeley Data Proteomic profile data of rabbit stem cell secretome and stem cell/tenocyte secretome and gene expression data, scratch assay and CAM assay data of tenocyte in vitro cultures supplemented with these two different secretomes - Mendeley Data and contains 2 sheets, called *without LPS and with LPS*, respectively. Both sheets are arranged the same. Therefore, only the first sheet *without LPS* is described. In column A, the plate number is defined. In column B, the label of the corresponding photo is shown. In column C, the area in µm^2^ is given. In column D, the length in µm is presented. In columns F-I, the same parameters are shown as in columns A-D. The same is true for columns K-M. Columns S-X exhibit calculations for means for the different rabbit donors (R7 for example is rabbit number 7); in addition the gap is calculated in % based on the areas measured. In columns AB-AG, the same is given as in columns S-X, just for another experimental condition (rbADSC Secretome w/o LPS). The same is true for columns AK-AP, respectively, where the condition is rbADSC:rbTenocytes Secretome w/o LPS.

### 3.5 CAM assay - angiogenesis

**File** DiB_Rabbit Secretomes_Figure 7.xlsx

This excel file is open access published in Mendeley Data Proteomic profile data of rabbit stem cell secretome and stem cell/tenocyte secretome and gene expression data, scratch assay and CAM assay data of tenocyte in vitro cultures supplemented with these two different secretomes - Mendeley Data and contains 8 sheets, called *Fig7A, Fig7B, Fig7C, Fig7D, Fig7E, Fig 7F, Fig7G, Fig7H* and *Fig7J*, respectively, according to the panels in the related research article Fig. 7 [1]. Sheet *Fig7A* contains data on eggs, with number of a specific egg in column A, days the egg survived in column B, to which group the egg belonged in column C, and the key for the abbreviations in columns D and E. The sheet *Fig7B* contains data on junctions expressed as fold change compared to day 0. The sheet is organized for three time points (columns B-D for day 2, columns E-G for day 4 and columns H-J for day 7), where for each time point the first of the three columns gives the value for the control, the second the value for the rbADSC secretome applied on the CAM and the third the value for the rbADSC/tenocyte secretome applied on the CAM. In lines 14 and 15 the mean and the standard deviation are calculated. Abbreviations are explained in lines 19 to 22. The sheet *Fig7C_F_I* contains data on sprouts expressed as fold change compared to day 0 and has otherwise the same structure as sheet *Fig7B* described above. The same is true for sheet *Fig7D* which contains data on total vessel length expressed as fold change compared to day 0; as well as for sheet *Fig7E* that contains data on mean vessel length, and sheet *Fig7G* containing data on vessel density. In sheet Fig7H, calculated angiogenic activity indices are given for the two secretomes, including junctions (line 2), total vessel length (line 3), vessel density (line 4) and sprouts hierarchy (line 5), resulting in the mean (line 7). Abbreviations are explained below in lines 11-13. Finally, the sheet Fig7J gives data on the CAM thickness expressed in µm for the three groups control (column B), rbADSC secretome (column C) and rbADSC/tenocyte secretome (column D) applied on the CAM surface.

## 4 Experimental Design, Materials and Methods

### 4.1 Secretomes harvested from rabbit ADSCs or from co-culture of rabbit ADSCs with tenocytes

Secretomes were harvested from either a pure rabbit adipose-derived stem cell culture or a rabbit ADSC/Achilles tenocyte co-culture as described in a previous publication [1]. Briefly, rabbit ADSCs were extracted from adipose tissue of female New Zealand White rabbits aged 5 to 7 months according to a protocol that was originally developed to extract ADSCs from human fat tissue [8]. As for the rabbit Achilles tenocytes, they were gained by collecting fresh Achilles tendons from female New Zealand White rabbits and cutting them in tiny pieces to allow the tenocytes migrate out of the tendon tissue in the Petri dish. While for expansion of the ADSCs DMEM-HG + 1 % P/S/G + 10 % FCS was used as culture medium, Ham’s F12 + 1 % P/S/G/A + 10 % FCS was used for tenocytes. For the co-culture, the two media were mixed in a ratio of 50 %:50 %. To prepare the cultures for secretome harvesting, cells of three rabbit donors were pooled [1].

### 4.2 CAM assay

The CAM assay was performed according to a previously published protocol where the two secretomes have been dropped on the CAM surface and angiogenic parameters assessed as reported earlier [1]. The angiogenic readouts included vessel density, number of junctions, total vessel length, mean vessel length, branch hierarchy and CAM thickness, utilizing ImageJ software (version 2.9.0, NIH, Bethesda, MD, USA) to measure and evaluate the photos of the CAM taken on days 0, 2, 4 and 7, respectively [1].

### 4.3 Angiogenic parameters and index

Angiogenic readouts were assessed and an angiogenic activity index (AAI) was calculated according to previously published procedures [1]. Briefly, the AAI that was originally developed in a comprehensive and standardized way for the chicken aortic ring assay [9], was used for the CAM assay in a slightly modified way, with less subcategories than applicable for the aortic ring, but not applicable for the CAM. Nevertheless, the AAI could be calculated according to (angiogenic readout at day 7 of control group minus value at day 0) subtracted from (angiogenic readout at day 7 of treatment group minus value at day 0) and this difference was divided by (angiogenic readout at day 7 of control group minus value at day 0) according to an equation published previously [1].

## Limitations

The CAM assay was performed for 7 days and images were taken at 0, 2, 4 and 7 days. One could extend the whole experiment to longer periods of time. Also, it would be possible to monitor angiogenesis in timely narrower intervals, i.e. daily taking photos and analyze corresponding parameters, which would strengthen the dynamic evaluation of angiogenesis.

## Ethics Statement

The study was conducted in accordance with the veterinary office of the Canton Zurich, Switzerland, with the ethical approval under licence No. 255/15.

## CRediT Author Statement

**Petra Wolint:** Conceptualization, Methodology, Data curation, Investigation, Writing – Original draft preparation, Writing – Reviewing and Editing. **Iris Miescher**: Conceptualization, Methodology, Data curation, Investigation, Writing – Original draft preparation, Writing – Reviewing and Editing. **Asma Mechakra**: Data analysis, Writing – Reviewing and Editing. **Patrick Jäger**: Data analysis, Writing – Reviewing and Editing. **Julia Rieber**: Data curation, Writing – Reviewing and Editing. **Maurizio Calcagni**: Supervision, Writing – Reviewing and Editing. **Pietro Giovanoli**: Supervision, Writing – Reviewing and Editing. **Viola Vogel**: Supervision, Writing – Reviewing and Editing. **Jess G. Snedeker**: Supervision, Writing – Reviewing and Editing. **Johanna Buschmann**: Conceptualization, Supervision, Writing – Original draft preparation, Writing – Reviewing and Editing, Fund raising, Administration.

## Data Availability

Mendeley DataProteomic profile data of rabbit stem cell secretome and stem cell/tenocyte secretome and gene expression data, scratch assay and CAM assay data of tenocyte in vitro cultures supplemented with these t (Original data).
